# The 15th Anniversary of *Viruses*: An Unwavering Commitment to Serving the Virology Community

**DOI:** 10.3390/v16040486

**Published:** 2024-03-22

**Authors:** Eric O. Freed

**Affiliations:** HIV Dynamics and Replication Program, Center for Cancer Research, National Cancer Institute, Frederick, MD 21702-1201, USA; efreed@mail.nih.gov

It is often said that two things in life are certain: death and taxes. Even without a professional interest in the subject, one might be tempted in today’s world to add a third: viruses.

As *Viruses* celebrates its 15th anniversary, this Editorial is being written against the background of a so-called ‘tripledemic’—the combination of influenza virus, SARS-CoV-2, and respiratory syncytial virus. This development has triggered an increase in hospital admissions of both the very young and the very old in Europe during the winter season [1]. However, despite how much we may know about viruses, there is always more to learn, there are always surprises around every corner, and there is always more work to be done in the fields of both primary research and public health management and communication.

During the past decade and a half, I have had the privilege of being Editor-in-Chief of *Viruses*, which was first launched in 2009 [2]. It is a role in which I take both professional pride and considerable pleasure, working on a strictly volunteer basis with the goal of creating a highly regarded and influential scientific journal that serves the continually evolving needs of the global virology community.

The field of virology has faced significant new challenges and has also witnessed exciting progress in recent decades. With a current impact factor of 4.7, *Viruses* itself has become a leader in the publication of high-quality virology papers. To date, the journal has published 13,690 papers, 5244 of which have been cited 10 times or more. *Viruses* has received an increased CiteScore of 7.1 according to the 2022 CiteScores™ released by Elsevier in June 2023. This places it in the top quartile (Q1) of the ‘Infectious Diseases’ category. This is a significant development, as many authors use this metric when deciding where to submit their work.

The success of *Viruses* as a well-recognized outlet for leading virology research is founded on the quality of its distinguished and highly committed editorial team, from the Editorial Board itself to the Reviewer Board to the editorial teams that prepare individual papers for publication. The journal has a broad scope that not only covers human and animal viruses but also insect, plant, and bacterial viruses. It is also very actively engaged in a number of outreach activities in the virology community, including conference organization, meeting sponsorships, early career investigator prizes, and travel awards, to name but a few. Every two years, we organize the *Viruses* conference to gather insights and exchange ideas with our peers in the field. The recent *Viruses* 2024 Conference, held in Barcelona, Spain, from 14 to 16 February 2024, was another significant milestone in this regard.

*Viruses* operates in accordance with the strictest ethical guidelines, and I am very proud of the journal that we have created in the past 15 years. We have an open and transparent communication policy, and we welcome all and any feedback and suggestions for improvement. It is no secret that MDPI, the publisher of *Viruses*, experienced certain reputational challenges in 2023, finding itself—together with several other open access publishers—falsely labeled as a ‘predatory publisher’ by an anonymous website. I am pleased to announce that this website has since been exposed as a ‘fake news’ source whose aim is to extort companies, as detailed in the following post from Cabells: https://blog.cabells.com/2024/01/16/unmasking-a-predator-predatoryreports-org/ (accessed on 5 March 2024).

Putting aside this genuinely predatory scam attempted against MDPI, certain valid concerns have been raised about the editorial practices of *Viruses*. These include the publication of too many Special Issues on topics that may be of limited interest; the use of reviewers who may lack the necessary expertise for the specific paper they are reviewing; and an over-emphasis on speed of production over quality of output.

These concerns need to be addressed, and I have in fact been pushing for this over the past couple of years. I can report that at our recent Editorial Board meeting in Barcelona, we had the opportunity to discuss how the Editorial Office has been working with great diligence over the past two years to improve the quality of peer review and Special Issues. I was made aware last November that the Directory of Open Access Journals (DOAJ) has introduced new criteria for creating Special Issues; according to these, new Special Issue Editors and Guest Editors must be approved by the Editor-in-Chief and Editorial Board Members. *Viruses* has been an early adopter of this procedure and has been operating with this workflow since 2022.

We will continue to make all the necessary and desirable process improvements as we continue to work towards our unwavering goal of providing researchers with reliable services that help alleviate the daily challenges of the academic world.

I expect *Viruses* to remain a journal universally recognized for its rapid and affordable publication of high-quality virology manuscripts from scientists around the world. Authors can expect streamlined and reliable processes, prompt and well-founded decisions, a swift turnaround time, and actions based on international best practices.

Together with all members of our Editorial Board, I look forward to continuing our journey with scholars worldwide, and I pledge to counter any unjustified or gratuitous criticism with prompt and proactive actions. We believe that scientists should be free to choose their preferred journal for sharing their research, and we extend a warm invitation to contact us about your own new research work in the critical field of virology. To celebrate the 15th anniversary of *Viruses*, I am editing a Special Issue that will contain articles from many of the world’s leading virologists. I cordially invite you to stay up to date with these proceedings.
